# Reduction of spermine synthase enhances autophagy to suppress Tau accumulation

**DOI:** 10.1038/s41419-024-06720-8

**Published:** 2024-05-13

**Authors:** Xianzun Tao, Jiaqi Liu, Zoraida Diaz-Perez, Jackson R. Foley, Ashley Nwafor, Tracy Murray Stewart, Robert A. Casero, R. Grace Zhai

**Affiliations:** 1 Department of Molecular and Cellular Pharmacology, University of Miami Miller School of Medicine, Miami, FL USA; 2 Sidney Kimmel Comprehensive Cancer Center, Johns Hopkins School of Medicine, Baltimore, MD USA

**Keywords:** Cellular neuroscience, Metabolomics

## Abstract

Precise polyamine metabolism regulation is vital for cells and organisms. Mutations in spermine synthase (SMS) cause Snyder–Robinson intellectual disability syndrome (SRS), characterized by significant spermidine accumulation and autophagy blockage in the nervous system. Emerging evidence connects polyamine metabolism with other autophagy-related diseases, such as Tauopathy, however, the functional intersection between polyamine metabolism and autophagy in the context of these diseases remains unclear. Here, we altered SMS expression level to investigate the regulation of autophagy by modulated polyamine metabolism in Tauopathy in *Drosophila* and human cellular models. Interestingly, while complete loss of *Drosophila spermine synthase* (*dSms*) impairs lysosomal function and blocks autophagic flux recapitulating SRS disease phenotype, partial loss of *dSms* enhanced autophagic flux, reduced Tau protein accumulation, and led to extended lifespan and improved climbing performance in Tauopathy flies. Measurement of polyamine levels detected a mild elevation of spermidine in flies with partial loss of *dSms*. Similarly, in human neuronal or glial cells, partial loss of SMS by siRNA-mediated knockdown upregulated autophagic flux and reduced Tau protein accumulation. Importantly, proteomics analysis of postmortem brain tissue from Alzheimer’s disease (AD) patients showed a significant albeit modest elevation of SMS level. Taken together, our study uncovers a functional correlation between polyamine metabolism and autophagy in AD: SMS reduction upregulates autophagy, suppresses Tau accumulation, and ameliorates neurodegeneration and cell death. These findings provide a new potential therapeutic target for AD.

## Introduction

Polyamines are positively charged alkylamines, including spermidine and spermine, and their precursor putrescine. Through interacting with negatively charged DNA, RNA, or proteins, they are broadly involved in cellular activities [1, 2]. Polyamines can be synthesized in the cells or accumulated in the extracellular environments. Mutations of polyamine metabolic enzymes or transporters are related to diseases [3–6]. The first identified polyamine pathway-related genetic disorder Snyder–Robinson Syndrome (SRS) is caused by mutations in *spermine synthase* (*SMS*), characterized by developmental delay, muscle/bone abnormalities, and intellectual disability [7]. Mechanistic studies showed that SMS deficiency results in the accumulation of spermidine and its metabolites [4, 8]. Increased byproducts of spermidine catabolism, aldehydes and ROS, damage membrane structures in the cells, such as mitochondria and lysosomes [4, 9]. Lysosomal damage leads to blocked autophagic flux [4].

The regulation of autophagy by polyamines has been broadly observed under diverse conditions, largely through modulating protein acetylation or eIF5A hypusination [10–20]. Interestingly, in contrast to the autophagy blockage effect of spermidine accumulation in SRS with complete loss of SMS, increasing cellular polyamine levels by either spermidine/spermine supplementation in wild-type or overexpression of ornithine decarboxylase 1 (ODC1) enhances autophagy [10, 11]. The underlying mechanism of these seemingly contradictory effects of polyamines on autophagy remains unclear, which hinders the potential of targeting the polyamine pathway to treat highly autophagy-related diseases, such as Tauopathy.

Tauopathy is a group of neurological disorders, including Alzheimer disease (AD), progressive supranuclear palsy, chronic traumatic encephalopathy, and so on, characterized by abnormal Tau accumulation in the brain [21–23]. Accumulated Tau species cause neuronal damage and death [24, 25]. Protein degradation machineries, including proteasome and autophagy, play important roles in Tau homeostasis [26–28]. Abnormal accumulation of intermediate autophagy structures is observed in the brains of AD patients or animal models, suggesting autophagic flux is blocked in AD [29–32]. Reducing neurotoxic Tau accumulation by repairing or enhancing autophagy is of strong potential to treat AD or other Tauopathies.

Polyamine metabolism appears to be a key pathway at the intersection of autophagy and Tauopathy. In addition to the above-mentioned roles of polyamines in autophagy regulation, studies have indicated the potential role of polyamines in Tauopathy [10, 13, 33–35]. Interestingly, similarly as in SRS, polyamine levels are upregulated in brains or plasma/serum of Tauopathy patients or animal models [33, 34, 36–41]. Manipulating polyamine pathway genes *Sat1* and *Azin2* has been shown to modulate Tauopathy progression in mouse models [33, 34]. Whether polyamine metabolism regulates Tauopathy through modulating autophagy is unclear.

In this study, we explored the interaction between SMS and autophagy under Tauopathy conditions. We revealed distinctive effects of heterozygous or homozygous loss-of-function *dSms* mutations in *Drosophila* and an unexpected protective effect of SMS reduction against Tauopathy in a *Drosophila* model and human cells. We further examined expression-level alterations of polyamine metabolism enzymes in AD brains and proposed SMS as a potential therapeutical target of Tauopathy.

## Materials and methods

### *Drosophila* stocks and genetics

Unless specified, flies were maintained on a cornmeal-molasses-yeast (62–106, Genesee Scientific, 15 g/L) medium at 25 °C, 65% humidity, 12 h light/12 h dark. The following fly strains were used in the studies: yw, actin-GAL4, elav-GAL4, repo-GAL4, UAS-lacZ, UAS-Tau, UAS-mCherry-GFP-Atg8a (Bloomington Drosophila Stock Center, all transgenes are in yw background); CG4300^e00382^ (*dSms* allele has a P element inserted in the opposite direction of the gene, The Exelixis Collection, Harvard Medical School, out-crossed to yw background). The SMS mutant chromosome and the transgenes were back-crossed into yw strains for three generations. The transgenes were recombined with the SMS mutant line in parallel.

### Fly climbing performance

Age-matched female or male flies from each genotype were placed in a vial marked with a line 8 cm from the bottom surface. The flies were gently tapped onto the bottom and given 10 s to climb. After 10 s, the number of flies that successfully climbed above the 8-cm mark was recorded and divided by the total number of flies. The assay was repeated ten times, and ten independent groups (a total of 100 flies) from each genotype were tested.

### Fly starvation resistance

Age-matched female or male flies were placed in a vial with three pieces of filter paper soaked with water; the filter paper was kept wet the entire process. The number of dead flies was counted every 2 h.

### Fly lifespan, brain staining, and immunoblot

These assays were done as described previously [9]. The following commercially available antibodies were used: anti-GABARAP for Drosophila Atg8a probing (PM037, MBL) [4], anti-Ref(2)P (ab178440, Abcam), anti-Tau (5A6, DSHB), anti-cleaved caspase 3 (9661, Cell Signaling), anti-LC3B (L7543, Sigma), anti-p62 (NBP1-48320, Novus Biologicals), anti-GFP (G5144, Invitrogen), anti-Actin (A1978, Sigma), Cy5-conjugated anti-HRP (123175021, Jackson ImmunoLab), and secondary antibodies conjugated to Alexa 488/568/647 (ThermoFisher Scientific) for brain immunohistochemical staining, or near-infrared (IR) dye 700/800 (Rockland) for western blot.

### Polyamine measurements

Samples were collected from flash-frozen flies stored at −80 °C. Polyamine content was determined by the pre-column dansylation, high-performance liquid chromatography method of Kabra et al. using 1,7 diaminoheptane as the internal standard [42]. The measurement shown here was done together with that shown in our previous publication [9]. The data of the control and *dSms*−/− flies are shared in these two studies.

### SMS knockdown in human cells

Co-transfections of control/SMS siRNA (4390843/s13173, ThermoFisher) and plasmids expressing EGFP (pEGFP-C1, Clontech) or Tau (pRK5- Tau, constructed in this study) into SH-SY5Y cells (CRL-2266, ATCC) was performed with JetPRIME transfection reagent (114-07, Polyplus) according to the manufacturer’s instruction in a 12-well plate. After 24 h, replace the medium, followed by another medium change after 48 h. On the fifth day, harvest the cells with RIPA buffer (R0278, Thermo) with proteinase inhibitors (11836170001, Roche) and phosphatase inhibitors (04906837001, Roche) on ice. The plasmid expressing Tau (pRK5- Tau) was subcloned from pRK5-EGFP-Tau (46904, Addgene) with the Tau coding sequence replacing the EGFP-Tau fusion sequence between the restriction sites ClaI and SalI.

### Tau K18 fibril labeling, transformation, and analysis

Tau K18 fibrils (NBP2-76793, NovusBio) were labeled with Alexa Fluor succinimidyl ester dye 647 (A37573, Thermo) and transformed into cells as reported [43]. Control or SMS siRNA are transfected into SVG p12 cells (CRL-8621, ATCC) on coverslips in 12-well plates as mentioned above. Three days after transfection, the sonicated Tau K18 fibrils were added into the medium. Two days later, the cells were fixed with 4% FA for 15 min, permeabilized with 0.4% Triton X-100 for 5 min and stained with p62 antibodies and DAPI. The cells were mounted on glass slides with VECTASHIELD Antifade Mounting Medium (Vector Laboratories) and kept at 4 °C until imaging on an Olympus IX81 confocal microscope. Images were processed with FluoView 10-ASW software (Olympus). Quantification of the fibril loci was performed using ImageJ program, as previously described [44]. Briefly, the fibril loci were segmented with interactive h-maxima watershed method and then the size and intensity of recognized loci were automatically measured with the particle analyzing tool.

### Statistics

Data were analyzed with Prism (GraphPad Software). Log-rank (Mantel–Cox) test with correction for multiple comparisons (Bonferroni method) was used for survival curve (lifespan) analysis. Student’s *t* test (two-tailed) was used for the comparison of two groups of samples for other assays. ANOVA multiple comparisons was used for assays with more than two groups. A *P* value smaller than 0.05 is considered statistically significant. *P < 0.05. **P < 0.01. ***P < 0.001.

## Results

### SMS reduction ameliorates Tauopathy in a *Drosophila* model

**Fig. 1:**
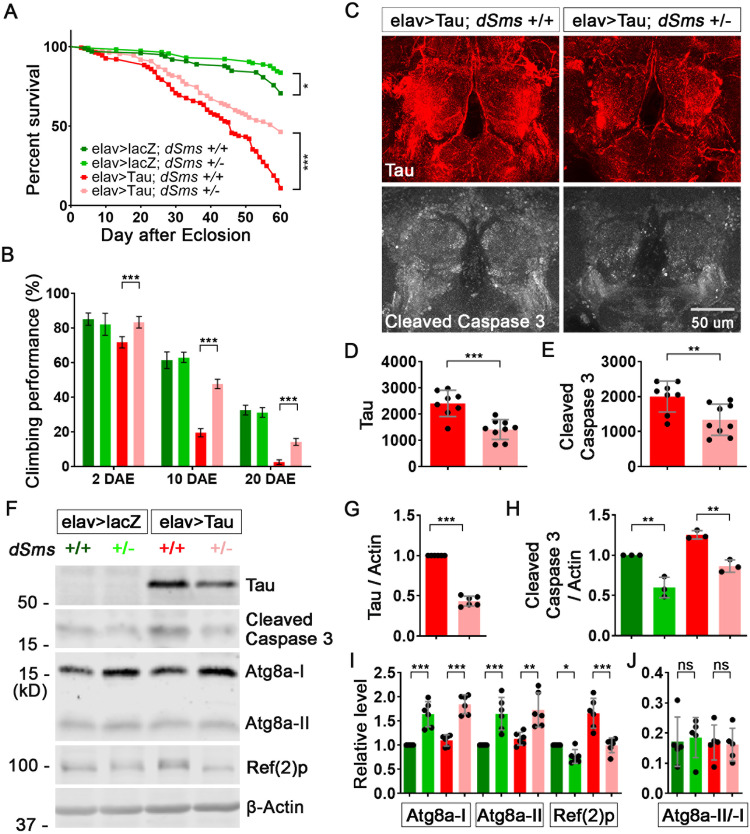
SMS reduction ameliorates Tauopathy in a *Drosophila* model. **A** Lifespan of female flies with indicated genotype. *n* = 99, 116, 164, 110; Log-rank (Mantel–Cox) test. **B** Climbing performance of female flies with indicated genotype at indicated ages. *n* = 100, 100, 100, 100. **C** Staining of brains of 10 DAE flies with antibodies against Tau or cleaved caspase 3. The image is a representative of multiple brains in each group, *n* = 8, 9. **D**, **E** Quantification of the staining signal intensity of Tau (**D**) or cleaved caspase 3 (**E**) in (**C**). *n* = 8, 9. **F** Western blot of Tau, cleaved caspase 3, autophagy marker Atg8a and Ref(2)p in heads of 10 DAE flies. The image is a representative of multiple experiments. **G**–**I** Quantification of the protein level of Tau (**G**), cleaved caspase 3 (**H**) or autophagy markers (**I**) in (**F**). All the protein levels were normalized with the β-Actin level. All the values were further normalized by that of the control flies. **J** The ratio of Atg8a-II/Atg8a-I in (**F**). *n* = 6, 3, 6, 6 (**G**–**J**). **B**, **H**–**J** Two-way ANOVA Sidak’s multiple comparisons. **D**, **E**, **G** Student’s *t* test. Data represent mean ± SEM.

The impact of polyamine catabolism on autophagy/lysosomal flux suggests that the polyamine metabolic pathway and protein homeostasis are closely connected [4, 9]. Given the importance of protein homeostasis in neurodegenerative Tauopathy, this connection suggests that polyamine metabolism might play a role in modulating disease progression in Tauopathy, where dysregulated protein homeostasis and autophagic flux are pathological hallmarks [29–32]. To test the effect of SMS reduction in Tauopathy, we established a *Drosophila* line with human Tau (hTau) expression and a heterozygous loss-of-function mutation of *dSms* (*dSms*^*+/−*^) [4, 24]. The mRNA expression level of SMS in *dSms*^*+/−*^ heterozygous flies was about half of that of wild-type [4]. As reported previously, compared to the control lacZ-expressing flies, hTau-expressing flies showed significantly reduced lifespan and impaired locomotor behavior [24] (Fig. 1A, B and Supplementary Fig. S1A, B). Interestingly, loss of one copy of SMS (*dSms*^*+/−*^) significantly extended the lifespan of hTau-expressing flies (Fig. 1A and Supplementary Fig. S1A) and improved the age-dependent behavior impairment (Fig. 1B and Supplementary Fig. S1B).

To dissect the cellular and molecular mechanisms underlying the beneficial effects of heterozygous loss of *dSms*, we first evaluated the level of hTau protein accumulation in fly brains. Staining the brains with antibodies against hTau proteins showed that hTau proteins were significantly reduced in the brains of *dSms*^+/−^ flies (Fig. 1C, D). Furthermore, the downstream neuronal toxicity effector of Tauopathy, cleaved caspase 3, significantly decreased in the brains of *dSms*^+/−^ flies (Fig. 1C, E). Western-blot analysis confirmed the decrease of hTau and cleaved caspase 3 in the heads of *dSms*^+/−^ flies (Fig. 1F–H). Of note, the mRNA level of hTau was not significantly altered in *dSms*^+/−^ brains (Supplementary Fig. S2), suggesting SMS reduction regulates hTau protein level post-transcriptionally.

Since Tau protein homeostasis is regulated by autophagy [43, 45, 46], we wondered whether autophagic flux is altered with partial loss of *dSms*. To evaluate autophagic flux, we first used several different autophagy markers, including Atg8a-I and its lipidated form, Atg8a-II, which are cytoplasmic and autophagosome-associated, respectively [47, 48]; and Ref(2)p, the cargo recruiter of autophagy, which is degraded along with the cargo in the functional autolysosome and could serve as an autophagic flux marker in combination with Atg8a [49]. Interestingly, Atg8a-I and its lipidated form, Atg8a-II were significantly upregulated, and Ref(2)p was significantly downregulated in *dSms*^*+/*−^ brains with either lacZ or hTau expression (Fig. 1F, I), suggesting that partial loss of *dSms* enhances autophagic flux independent of hTau expression. Notably, the ratios of Atg8a-II to Atg8a-I were not significantly changed in *dSms*^*+/−*^ brains (Fig. 1J), suggesting SMS is unlikely to be involved in autophagosome maturation. The enhanced autophagic flux with partial loss of *dSms* could be the underlying mechanism of the lifespan-extending effect of heterozygous loss of *dSms* in either lacZ or hTau-expressing flies (Fig. 1A and Supplementary Fig. S1A). Collectively, these results show that partial loss of SMS enhances autophagic flux, extends lifespan, and ameliorates neurodegenerative phenotypes in Tauopathy models.

### SMS regulates autophagy in a biphasic manner

**Fig. 2:**
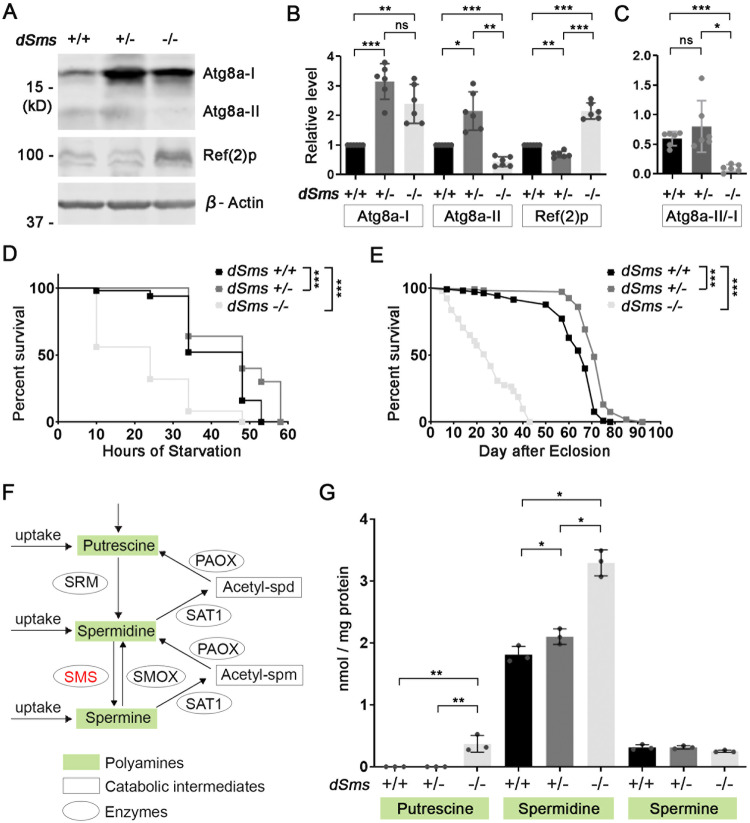
SMS regulates autophagy in a biphasic manner. **A** Western blot of autophagy marker Atg8a and cargo recruiter Ref(2)p in 5 DAE flies. The image is a representative of multiple separate experiments. **B** Quantification of the protein level of Atg8a-I, Atg8a-II, and Ref(2)p in (**A**). All the protein levels were normalized with the β-Actin level. All the values were further normalized by that of the control flies. **C** The ratio of Atg8a-II/Atg8a-I in (**A**). *n* = 6; one-way ANOVA Tukey’s multiple comparisons. **D** Survival curve of 10 DAE female flies with indicated genotype under starvation. *n* = 50, 50, 25; Log-rank (Mantel–Cox) test. **E** Lifespan of female flies with indicated genotype. *n* = 105, 107, 91; Log-rank (Mantel–Cox) test. **F** Diagram of the polyamine metabolism pathway. **G** Polyamine levels of 10 DAE female flies with indicated genotype. Each dot indicates a sample of a homogenized mixture of ten flies. *n* = 3; one-way ANOVA Tukey’s multiple comparisons. Data represent mean ± SEM. The measurement showed here was done together with that showed in our previous publication [9]. The data of the control and *dSms* −/− flies are shared in these two studies.

The neuroprotective effects observed in these dSMS heterozygotes are in contrast to our previous observations in dSMS homozygous mutants, where complete loss of dSMS (*dSms*^−/−^*)* resulted in lysosomal dysfunction and impaired autophagic flux [4]. To dissect the underlying mechanisms and exclude the possible influence by lacZ or hTau expression, we compared the autophagy level in flies with heterozygous or homozygous *dSms* loss without lacZ or hTau overexpression (genotype: heterozygous, *dSms*^+/−^; homozygous, *dSms*^−/−^). Strikingly, autophagic flux alteration in heterozygous or homozygous flies occurred in opposite directions: while Atg8a-I (cytoplasmic) was increased in either homozygous or heterozygous flies, Atg8a-II (autophagosome-associated) was reduced in homozygous flies but increased in heterozygous flies (Fig. 2A, B), leading to significantly reduced ratio of Ata8a-II to Atg8a-I in homozygous flies but not in heterozygous flies (Fig. 2C). In addition, the autophagy cargo recruiter Ref(2)p was accumulated in homozygous flies but reduced in heterozygous flies (Fig. 2A, B). Taken together, these data suggest that autophagic flux is blocked in homozygous flies, as reported in our previous study, but is upregulated in heterozygous flies.

To further assess the functional consequence of autophagy alteration, we evaluated the starvation resistance of these flies, which is highly dependent on autophagy activity. While homozygous flies died earlier under starvation compared to control flies, heterozygous flies survived longer than control flies (Fig. 2D and Supplementary Fig. S3A). We then determined the lifespan of these flies, which is also related to autophagy activity. While homozygous flies have a reduced lifespan as reported [4], heterozygous flies lived significantly longer than control flies (Fig. 2E and Supplementary Fig. S3B).

We next questioned whether the different autophagic activity alteration in heterozygous and homozygous flies results from different polyamine level alteration in these flies. To test the possibility, we measured the polyamine levels in these flies (Fig. 2F). Interestingly, the level of spermidine is elevated in either homozygous or heterozygous flies compared to that in the control flies (Fig. 2G and Supplementary Fig. S3C). But the spermidine level increase in heterozygous flies is much milder than that in homozygous flies (Fig. 2G and Supplementary Fig. S3C). The spermine level is reduced in homozygous flies, as expected, but not altered in heterozygous flies (Fig. 2G and Supplementary Fig. S3C). Collectively, these data suggest that SMS regulates autophagy through modulating spermidine level. Specifically, high level of spermidine accumulation in complete loss of *dSms* impairs autophagic flux but mild spermidine elevation in partial loss of *dSms* upregulates autophagic flux.

To evaluate the effect of the combination of polyamine supplementation and SMS reduction, we fed spermidine to *dSms*^*+/+*^ or *dSms*^*−/−*^ flies with lacZ or hTau overexpression. While 1 mM of spermidine feeding slightly extends the median survival of *dSms*^*+/+*^ flies with lacZ or hTau overexpression, it significantly shortens the median survival and lifespan of *dSms*^*−/−*^ flies with hTau overexpression (Supplementary Fig. S4), which could mimic the toxicity of spermidine overdose under SRS condition.

### SMS reduction upregulates autophagic flux in both neurons and glia in *Drosophila*

**Fig. 3:**
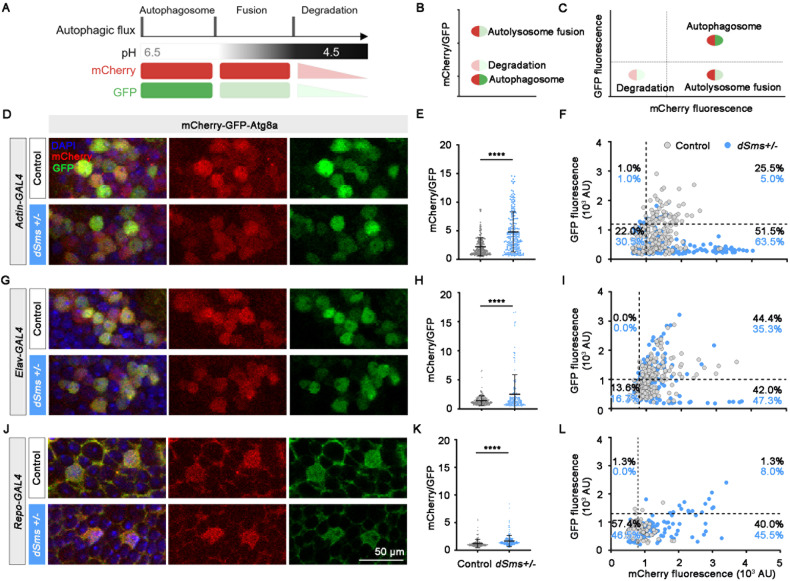
SMS reduction upregulates autophagic flux in both neurons and glia in *Drosophila.* **A** Diagram of the fluorescence signals from the reporter protein mCherry-GFP-Atg8a under different conditions. **B** The ratio of total fluorescence intensity of mCherry to GFP in single cells indicates the enrichment of a specific stage of autophagic flux, which can be used to evaluate the average autophagic flux level in a cell. **C** Scat plotting of mCherry and GFP fluorescence intensity divides cells into subgroups enriched with specific stage of autophagic flux. **D**, **G**, **J** Images of brain cells of control or *dSms*^*+/−*^ flies with *actin*-, *elav*-, or *repo*-driven mCherry-GFP-Atg8a expression, 10 DAE, female flies. **E**, **H**, **K** The ratio of total fluorescence intensity of mCherry to GFP in single cells in the brains from (**D**, **G**, **J**), respectively. **F**, **I**, **L** Grouping cells with the florescence ratio in single cells from (**D**, **G**, **J**), respectively. Each dot represents a single cell. In total, 50 cells from each brain, 3 or 4 brains from each group, were measured. *n* = 150 or 200; Student’s *t* test. Data represent mean ± SEM.

To further assess the regulation of SMS on autophagic flux in single cells in vivo, we used an Atg8a reporter tagged with mCherry and pH-sensitive GFP [50] to monitor autophagic flux (Fig. 3A). In neutral pH environment, both mCherry and GFP fluorescence, in the cytosol or on the autophagosome membrane, are stable. However, after fusion with acidic lysosomes, the pH decrease causes a reduction in GFP fluorescence. As autolysosomes mature, the reporter proteins start to be degraded, as indicated by mCherry fluorescence reduction (Fig. 3A). As such, the intensity and the ratio of the two fluorescence signals can be used to differentiate autophagic structures at different steps of the autophagic flux. In phagophores or autophagosomes, both mCherry and GFP signals are high. In newly formed autolysosomes, the mCherry signal is high, and the GFP signal is low. In matured autolysosomes, the mCherry signal is medium, and the GFP signal is low. The ratio of mCherry to GFP fluorescence within a single cell, serves as a proxy to the autophagic flux in the cell: a higher ratio indicates more functional autolysosome enrichment (Fig. 3B). Based on the fluorescence intensity of mCherry and GFP in a scatter plot, cells can be divided into three populations: high-mCherry/high-GFP: cells enriched with phagophores/autophagosomes or free reporter proteins; high-mCherry/low-GFP: cells enriched with functional autolysosomes; and low-mCherry/low-GFP, cells with matured autolysosome enriched or low expression of the reporter proteins (Fig. 3C). Using this reporter system, we showed that the autophagic flux is blocked in homozygous *dSms*^−/−^ mutant brains [4].

To uncover potential cell type-specific alterations in autophagy in the brain, we established *Drosophila* lines expressing the mCherry-GFP-Atg8a reporter controlled by different drivers (*actin-GAL4* for ubiquitous expression, *elav-GAL4* for neuronal-specific expression and *repo-GAL4* for glial expression), with or without partial loss of *dSms* (Fig. 3D, J). Interestingly, we found that regardless of the cell types of reporter expression, the ratio of mCherry to GFP fluorescence in the brain cells of the flies with heterozygous loss of *dSms* is significantly higher than that of the control flies (Fig. 3E, H, K), suggesting SMS reduction upregulates autophagic flux in both neurons and glia. Notably, the general intensity of mCherry or GFP signals in brain cells of *dSms*^+/−^ heterozygous flies is lower than that of control flies (Fig. 3D, G, J), which may indicate a potentially higher autophagic flux in the heterozygous flies, although the possibility of a lower expression level of the reporter protein could not be completely excluded. The populations of cells with high-mCherry/low-GFP in *dSms*^*+/−*^ heterozygous flies are significantly larger than that in control flies in all three cell type-specific expressing conditions (Fig. 3F, I, L), suggesting an increase of functional autolysosomes in all cells with partial loss of SMS. Notably, we found that compared to the neuronal population (driven by elav-GAL4), glial cells (driven by repo-GAL4) have a significantly larger low-mCherry/low-GFP cell population but smaller high-mCherry/high-GFP cell population (Fig. 3I, L). This might indicate a higher rate of matured autolysosomes in glia and/or less accumulated reporter proteins because of a shorter lifespan of glia. Taken together, SMS reduction upregulates autophagic flux in both neurons and glia, even though the autophagic flux itself might be different in the two types of cells.

### SMS knockdown reduces Tau accumulation in human neuronal and glial cell lines

**Fig. 4:**
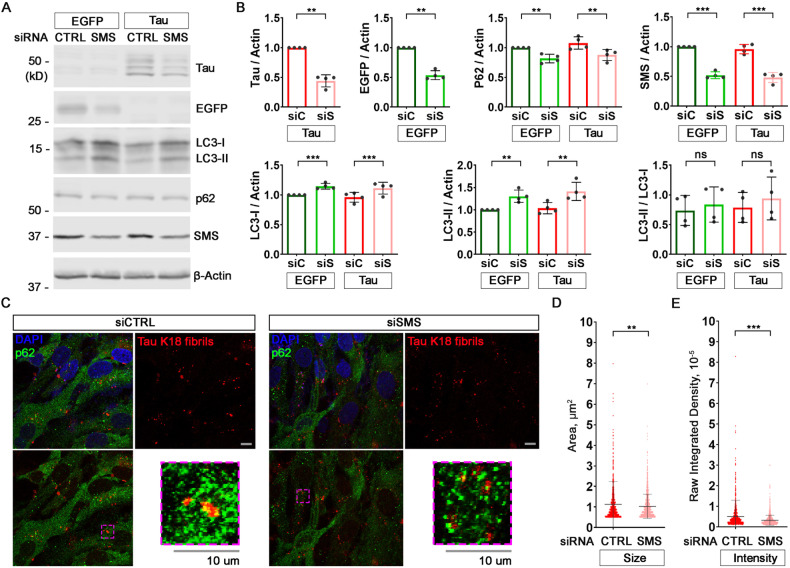
SMS knockdown upregulates autophagy and suppresses Tau accumulation in human neuronal or glial cell lines. **A** Western blot of Tau, EGFP, autophagy marker LC3, cargo recruiter p62 and SMS in SH-SY5Y cells with Tau/EGFP plasmids and Control/SMS siRNA transfection. The image is a representative of four separate experiments. **B** Quantification of the protein levels of Tau (5A6), EGFP, LC3-I (cytoplasmic), LC3-II (autophagosome-associated), p62, and SMS in (**A**). All the protein levels were normalized with the β-Actin level. All the values were further normalized by that of the control cells. *n* = 4; Student’s *t* test (Tau or EGFP) or two-way ANOVA Sidak’s multiple comparisons (others). **C** p62 staining and Alexa 647-conjugated Tau K18 fibrils in SVG p12 cells with Control/SMS siRNA transfection. The images are representatives of five fields. **D**, **E** Quantification of the size (area) (**D**) and intensity (**E**) of the Alexa 647-conjugated Tau K18 fibrils in (**C**). *n* = 5; Student’s *t* test. Data represent mean ± SEM.

To examine whether the effect of partial loss of SMS on autophagy and Tau accumulation is conserved between *Drosophila* and human, we then tested the effect of SMS reduction in human cells. SMS knockdown with siRNA in neuron-like SH-SY5Y cells mildly upregulated LC3-I and LC3-II (the human homologs of *Drosophila* Atg8a-I and Atg8a-II), downregulated p62 (the human homolog of *Drosophila* Ref(2)p [49]) and significantly decreased exogenous Tau protein accumulation (Fig. 4A, B). Consistent with the observation on Atg8a-II/tg8a-I ratios in flies, the ratios of LC3-II to LC3-I were not significantly changed with SMS knockdown, again suggesting SMS doesn’t affect autophagosome maturation. Notably, overexpressed EGFP was also significantly downregulated by SMS knockdown, accompanied with LC3 upregulation and p62 downregulation (Fig. 4A, B), suggesting the effect of SMS knockdown on autophagy is not Tau-specific but rather a general effect, which is consistent with the observation of the effect of heterozygous loss of *dSms* on autophagy in vivo in flies (Figs. 1 and 2).

To validate the mild spermidine accumulation effect of SMS reduction, we measured the polyamine levels in the heads of *dSms* heterozygous flies with lacZ or hTau overexpression and in SH-SY5Y cells with SMS knockdown and EGFP or Tau overexpression (Supplementary Fig. S5). Spermidine levels are increased in dSms heterozygous flies expressing either lacZ or hTau (Supplementary Fig. S5A, B). While putrescine or spermine levels did not show consistent trends of change (Supplementary Fig. S5C), spermidine levels in SMS knockdown SH-SY5Y cells expressing either EGFP or Tau showed a trend of increase, although not statistically significant, likely due to the variation between different experiments (Supplementary Fig. S5C, D). Collectively, increased levels of spermidine detected in partial loss of SMS cells overexpressing Tau suggest mild spermidine accumulation as a potential driving force mediating the protective effects of partial loss of SMS.

Given that glial cells have been shown to play important roles in Tau aggregate regulation [51, 52], we next investigated the effect of SMS reduction on Tau aggregates in human glial cells using a Tau K18 fibrils uptake assay [43]. We incubated control or SMS siRNA-knockdown SVG p12 cells with Tau K18 fibrils conjugated with Alexa-fluor-647. Consistent with the previous report [43], Tau K18 fibrils were taken up by SVG p12 cells efficiently (Fig. 4C). In addition, we observed colocalization of Tau K18 fibrils and p62 (Fig. 4C), suggesting the regulation of Tau fibrils by autophagy. As shown in Fig. 4C–E, SMS knockdown significantly decreased the size and the intensity of Tau fibril loci in SVG p12 cells. This result is consistent with the observation of reduction of Tau accumulation with partial loss of SMS in both human neuronal SH-SY5Y cells and *Drosophila* in vivo models.

### SMS levels are elevated in postmortem AD patient brains

**Fig. 5:**
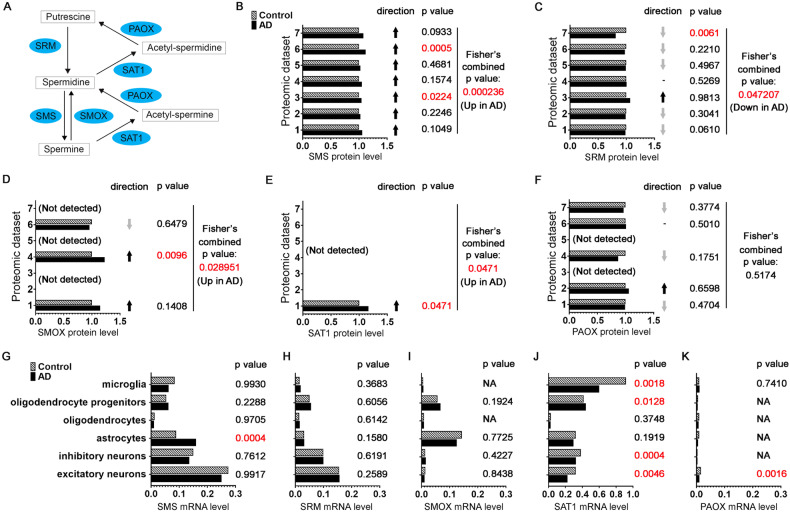
SMS expression level is upregulated in postmortem AD patient brains. **A** Diagram of the polyamine pathway with the enzymes highlighted. **B**–**F** The protein levels of SMS (**B**), SRM (**C**), SMOX (**D**), SAT1 (**E**), or PAOX (**F**) in the frontal cortex of control or AD patient brains measured by quantitative mass spectrometry from seven published datasets with normal distribution pattern [53–57]. The one-tailed *t* test *P* values were combined with Fisher’s method. **G**–**K** The mRNA levels of SMS (**G**), SRM (**H**), SMOX (**I**), SAT1 (**J**), or PAOX (**K**) in indicated brain cells from the prefrontal cortex of control or AD patients measured by single-nucleus RNA seq reported recently [62]. The *P* values were obtained with the Poisson mixed model.

To directly assess the potential correlation of SMS and polyamine metabolism with AD, we examined the expression level of SMS and other polyamine pathway enzymes (Fig. 5A) in the frontal cortex of AD patient brains using a meta-analysis [53] of seven published proteomic datasets based on the TMT-LC/LC-MS/MS platform [54–57]. The protein level of SMS is consistently upregulated in trend in AD brains in all seven datasets, and the combined *P* value analysis shows the upregulation is significant (Fig. 5B). In contrast, the trend of the change of the protein level of spermidine synthase (SRM) in AD brains varies among the datasets, and the combined analysis suggests it is downregulated in AD brains (Fig. 5C). The protein levels of the polyamine catabolic enzymes are under the detection limit in some datasets and the alterations vary among the datasets (Fig. 5D–F). However, combined analysis shows that both spermine oxidase (SMOX) and spermidine/spermine acetyltransferase (SAT1) are upregulated in AD brains. Collectively, it suggests that spermidine and spermine interconversions mediated by SMS and spermine catabolic enzymes are elevated in AD, which likely results in the accumulation of potentially toxic metabolites and increased oxidative stress in the brain as observed in some cancer or neurodegeneration conditions [58–61].

To further uncover potential cell type-specific changes of the expression level of SMS and other polyamine pathway enzymes in the brain, we analyzed a published single-nucleus RNA seq dataset from prefrontal cortex samples of control or AD patients [62]. Interestingly, the mRNA level of SMS is significantly upregulated in astrocytes of AD patients but is largely unchanged in other cell types (Fig. 5G). As a comparison, the mRNA level of SRM is not significantly altered in all the measured cell types (Fig. 5H). The mRNA levels of polyamine catabolic enzymes are not significantly changed in astrocytes of AD patients (Fig. 5I–K), further highlighting the specificity of SMS upregulation in AD.

## Discussion

In this study, we investigated the regulation of SMS on autophagy in physiological or disease conditions (Figs. 1–3). We found that heterozygous loss-of-function of *dSms* significantly upregulates autophagy, decreases hTau protein accumulation, and ameliorates Tauopathy in a *Drosophila* model (Fig. 1). Consistently, SMS knockdown in human neuronal or glial cells also enhances autophagic activity and decreases exogenous Tau accumulation (Fig. 4). Moreover, we showed that SMS, together with some polyamine catabolic enzymes, is elevated in AD patient brains (Fig. 5), suggesting SMS plays roles in AD and could be a therapeutic target.

**Fig. 6:**
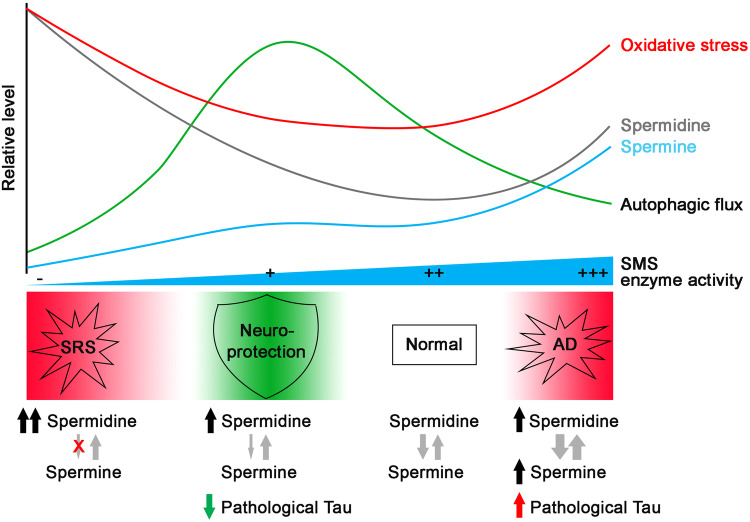
Model of the interaction of polyamine pathway and autophagy in SRS and Tauopathy. Under SRS conditions, with complete loss of SMS, there is a significant increase in spermidine and a decrease in spermine levels, which leads to an overall increase in total polyamines [4, 9]. Conversely, under AD conditions, with increased SMS and the conversion of spermine to spermidine, both spermidine and spermine levels increase [36–41]. The elevated polyamines and their metabolism in these two pathological conditions result in higher levels of oxidative stress [68–70] and impaired autophagic flux [29–32]. However, a partial reduction of SMS leads to a mild accumulation of spermidine without significant changes in spermine levels, which avoids triggering oxidative stress and enhances autophagic flux. While impaired autophagic flux in AD aggravates pathological Tau accumulation, enhancing autophagic flux through SMS partial reduction promotes Tau clearance and confers neuroprotection.

Our previous studies in flies with homozygous loss-of-function mutation of *dSms* modeling SRS discovered the detrimental effect of accumulated spermidine on autophagic flux, caused by increased aldehyde-mediated lysosomal damage [4, 9]. This observation is in contrast to the reported beneficial effect of polyamine supplementation on autophagy tested in multiple organisms [10, 13]. In this study, we found opposing effects of partial versus complete loss of *dSms* on autophagic activity (Fig. 2), which provides a possible explanation to the “controversy” between accumulated spermidine in SRS and that from polyamine supplementation. It is likely that increased spermidine enhances autophagy gene expression in both conditions (Fig. 2A, B). However, the higher level of spermidine accumulation results in abnormal accumulation of the catabolic byproducts, including aldehydes and ROS, and therefore causes lysosomal damage and the subsequent block of autophagic flux in SRS [4, 9] (Fig. 6).

The observation of elevated polyamine levels in AD patient brains or plasma [36–41] (Fig. 6) also raises questions on the perceived beneficial effect of spermidine supplementation on brain health in human or animal models [11, 20, 63–67]. Different levels of polyamine metabolic activity might contribute to this discrepancy. Consistent with the upregulation of polyamine metabolism enzymes in AD patient brains (Fig. 4), the polyamine pathway in Tauopathy animal models is activated [33, 34]. It is likely that high level of polyamine catabolism, such as SMOX-mediated spermine back-conversion to spermidine, results in oxidative stress in Tauopathy [68–70], whose detrimental effect overcomes the beneficial effect of polyamine themselves (Fig. 6). An optimal level of exogenous polyamine supplementation might be able to assert the beneficial effect of polyamines without triggering the activation of the polyamine catabolic pathway, due to moderate levels of cellular accumulation. It would be interesting to test the effects of the combination of polyamine supplementation and inhibition of polyamine catabolism on Tauopathy.

The cellular process of polyamines regulating autophagy has been broadly studied in diverse conditions [71]. The reported mechanisms vary in different cell types or physiological contexts. Among them, post-translational modifications, including histone and non-histone acetylation and eIF5A hypusination, play key roles in aging and neurological function-related autophagy/mitophagy regulation [10, 13, 16, 17, 19, 20, 66]. Our study here established the correlation of mild accumulation of spermidine and autophagy enhancement, suggesting potential new mechanisms of polyamine and autophagy regulation. Examining protein acetylation and eIF5A hypusination levels in different tissues of our fly models, including *dSms* heterozygous and homozygous flies, could reveal interesting cellular insights. In addition to regulating autophagy, polyamines modulate cell or organism function through other mechanisms, such as regulating general or specific gene expression [72, 73], circadian clock [74], and mitochondrial biogenesis or activity [75, 76]. The functional contribution of these non-autophagy mechanisms to the beneficial effect of mild spermidine accumulation by SMS partial reduction remains to be explored.

In summary, here we report that SMS regulates autophagic activity through modulating the level of polyamines and the catabolic process. While severe to complete loss or overexpression of SMS causes significant polyamine dysregulation and autophagy blockade, partial reduction of SMS leads to mildly accumulated spermidine and enhanced autophagic activity, promotes Tau clearance and confers neuroprotection. Our discovery suggests SMS as a potential therapeutical target for AD.

### Supplementary information

Supplementary figures

Original western blots

## Data Availability

All data are available in the main text or the supplementary materials.

## References

[1] Madeo F, Eisenberg T, Pietrocola F, Kroemer G (2018). Spermidine in health and disease. Science.

[2] Pegg AE (2016). Functions of polyamines in mammals. J Biol Chem.

[3] van Veen S, Martin S, Van den Haute C, Benoy V, Lyons J, Vanhoutte R (2020). ATP13A2 deficiency disrupts lysosomal polyamine export. Nature.

[4] Li C, Brazill JM, Liu S, Bello C, Zhu Y, Morimoto M (2017). Spermine synthase deficiency causes lysosomal dysfunction and oxidative stress in models of Snyder-Robinson syndrome. Nat Commun.

[5] Rodan LH, Anyane-Yeboa K, Chong K, Klein Wassink-Ruiter JS, Wilson A, Smith L (2018). Gain-of-function variants in the ODC1 gene cause a syndromic neurodevelopmental disorder associated with macrocephaly, alopecia, dysmorphic features, and neuroimaging abnormalities. Am J Med Genet A.

[6] Bupp CP, Schultz CR, Uhl KL, Rajasekaran S, Bachmann AS (2018). Novel de novo pathogenic variant in the ODC1 gene in a girl with developmental delay, alopecia, and dysmorphic features. Am J Med Genet A.

[7] Cason AL, Ikeguchi Y, Skinner C, Wood TC, Holden KR, Lubs HA (2003). X-linked spermine synthase gene (SMS) defect: the first polyamine deficiency syndrome. Eur J Hum Genet.

[8] Abela L, Simmons L, Steindl K, Schmitt B, Mastrangelo M, Joset P (2016). N(8)-acetylspermidine as a potential plasma biomarker for Snyder-Robinson syndrome identified by clinical metabolomics. J Inherit Metab Dis.

[9] Tao X, Zhu Y, Diaz-Perez Z, Yu SH, Foley JR, Stewart TM (2022). Phenylbutyrate modulates polyamine acetylase and ameliorates Snyder-Robinson syndrome in a Drosophila model and patient cells. JCI Insight.

[10] Eisenberg T, Knauer H, Schauer A, Buttner S, Ruckenstuhl C, Carmona-Gutierrez D (2009). Induction of autophagy by spermidine promotes longevity. Nat Cell Biol.

[11] Gupta VK, Scheunemann L, Eisenberg T, Mertel S, Bhukel A, Koemans TS (2013). Restoring polyamines protects from age-induced memory impairment in an autophagy-dependent manner. Nat Neurosci.

[12] Eisenberg T, Abdellatif M, Schroeder S, Primessnig U, Stekovic S, Pendl T (2016). Cardioprotection and lifespan extension by the natural polyamine spermidine. Nat Med.

[13] Morselli E, Marino G, Bennetzen MV, Eisenberg T, Megalou E, Schroeder S (2011). Spermidine and resveratrol induce autophagy by distinct pathways converging on the acetylproteome. J Cell Biol.

[14] Minois N, Carmona-Gutierrez D, Bauer MA, Rockenfeller P, Eisenberg T, Brandhorst S (2012). Spermidine promotes stress resistance in Drosophila melanogaster through autophagy-dependent and -independent pathways. Cell Death Dis.

[15] Yang Y, Chen SC, Zhang YQ, Lin XX, Song YY, Xue ZL (2017). Induction of autophagy by spermidine is neuroprotective via inhibition of caspase 3-mediated Beclin 1 cleavage. Cell Death Dis.

[16] Pietrocola F, Lachkar S, Enot DP, Niso-Santano M, Bravo-San Pedro JM, Sica V (2015). Spermidine induces autophagy by inhibiting the acetyltransferase EP300. Cell Death Differ.

[17] Yue F, Li W, Zou J, Jiang X, Xu G, Huang H (2017). Spermidine prolongs lifespan and prevents liver fibrosis and hepatocellular carcinoma by activating MAP1S-mediated autophagy. Cancer Res.

[18] Lubas M, Harder LM, Kumsta C, Tiessen I, Hansen M, Andersen JS (2018). eIF5A is required for autophagy by mediating ATG3 translation. EMBO Rep.

[19] Zhang H, Alsaleh G, Feltham J, Sun Y, Napolitano G, Riffelmacher T (2019). Polyamines control eIF5A hypusination, TFEB translation, and autophagy to reverse B cell senescence. Mol Cell.

[20] Liang Y, Piao C, Beuschel CB, Toppe D, Kollipara L, Bogdanow B (2021). eIF5A hypusination, boosted by dietary spermidine, protects from premature brain aging and mitochondrial dysfunction. Cell Rep.

[21] Shi Y, Zhang W, Yang Y, Murzin AG, Falcon B, Kotecha A (2021). Structure-based classification of tauopathies. Nature.

[22] Kosik KS, Joachim CL, Selkoe DJ (1986). Microtubule-associated protein tau (tau) is a major antigenic component of paired helical filaments in Alzheimer disease. Proc Natl Acad Sci USA.

[23] Wood JG, Mirra SS, Pollock NJ, Binder LI (1986). Neurofibrillary tangles of Alzheimer disease share antigenic determinants with the axonal microtubule-associated protein tau (tau). Proc Natl Acad Sci USA.

[24] Wittmann CW, Wszolek MF, Shulman JM, Salvaterra PM, Lewis J, Hutton M, et al. Tauopathy in Drosophila: neurodegeneration without neurofibrillary tangles. Science. 2001;293:711–4. 10.1126/science.1062382 11408621

[25] Santacruz K, Lewis J, Spires T, Paulson J, Kotilinek L, Ingelsson M.et al. Tau suppression in a neurodegenerative mouse model improves memory function. Science. 2005;309:476–81. 10.1126/science.1113694 PMC1574647 16020737

[26] Hamano T, Gendron TF, Causevic E, Yen SH, Lin WL, Isidoro C (2008). Autophagic-lysosomal perturbation enhances tau aggregation in transfectants with induced wild-type tau expression. Eur J Neurosci.

[27] Berger Z, Ravikumar B, Menzies FM, Oroz LG, Underwood BR, Pangalos MN (2006). Rapamycin alleviates toxicity of different aggregate-prone proteins. Hum Mol Genet.

[28] Myeku N, Clelland CL, Emrani S, Kukushkin NV, Yu WH, Goldberg AL (2016). Tau-driven 26S proteasome impairment and cognitive dysfunction can be prevented early in disease by activating cAMP-PKA signaling. Nat Med.

[29] Nixon RA, Wegiel J, Kumar A, Yu WH, Peterhoff C, Cataldo A (2005). Extensive involvement of autophagy in Alzheimer disease: an immuno-electron microscopy study. J Neuropathol Exp Neurol.

[30] Bordi M, Berg MJ, Mohan PS, Peterhoff CM, Alldred MJ, Che S (2016). Autophagy flux in CA1 neurons of Alzheimer hippocampus: Increased induction overburdens failing lysosomes to propel neuritic dystrophy. Autophagy.

[31] Lee JH, Yu WH, Kumar A, Lee S, Mohan PS, Peterhoff CM (2010). Lysosomal proteolysis and autophagy require presenilin 1 and are disrupted by Alzheimer-related PS1 mutations. Cell.

[32] Sanchez-Varo R, Trujillo-Estrada L, Sanchez-Mejias E, Torres M, Baglietto-Vargas D, Moreno-Gonzalez I (2012). Abnormal accumulation of autophagic vesicles correlates with axonal and synaptic pathology in young Alzheimer’s mice hippocampus. Acta Neuropathol.

[33] Sandusky-Beltran LA, Kovalenko A, Placides DS, Ratnasamy K, Ma C, Hunt JB (2021). Aberrant AZIN2 and polyamine metabolism precipitates tau neuropathology. J Clin Investig.

[34] Sandusky-Beltran LA, Kovalenko A, Ma C, Calahatian JIT, Placides DS, Watler MD (2019). Spermidine/spermine-N(1)-acetyltransferase ablation impacts tauopathy-induced polyamine stress response. Alzheimers Res Ther.

[35] Polis B, Karasik D, Samson AO (2021). Alzheimer’s disease as a chronic maladaptive polyamine stress response. Aging.

[36] Inoue K, Tsutsui H, Akatsu H, Hashizume Y, Matsukawa N, Yamamoto T (2013). Metabolic profiling of Alzheimer’s disease brains. Sci Rep.

[37] Mahajan UV, Varma VR, Griswold ME, Blackshear CT, An Y, Oommen AM (2020). Dysregulation of multiple metabolic networks related to brain transmethylation and polyamine pathways in Alzheimer disease: a targeted metabolomic and transcriptomic study. PLoS Med.

[38] Morrison LD, Kish SJ (1995). Brain polyamine levels are altered in Alzheimer’s disease. Neurosci Lett.

[39] Graham SF, Chevallier OP, Elliott CT, Holscher C, Johnston J, McGuinness B (2015). Untargeted metabolomic analysis of human plasma indicates differentially affected polyamine and L-arginine metabolism in mild cognitive impairment subjects converting to Alzheimer’s disease. PLoS ONE.

[40] Sternberg Z, Podolsky R, Nir A, Yu J, Nir R, Halvorsen SW (2022). Elevated spermidine serum levels in mild cognitive impairment, a potential biomarker of progression to Alzheimer dementia, a pilot study. J Clin Neurosci.

[41] Wortha SM, Frenzel S, Bahls M, Habes M, Wittfeld K, Van der Auwera S (2023). Association of spermidine plasma levels with brain aging in a population-based study. Alzheimers Dement.

[42] Kabra PM, Lee HK, Lubich WP, Marton LJ (1986). Solid-phase extraction and determination of dansyl derivatives of unconjugated and acetylated polyamines by reversed-phase liquid chromatography: improved separation systems for polyamines in cerebrospinal fluid, urine and tissue. J Chromatogr.

[43] Kolay S, Vega AR, Dodd DA, Perez VA, Kashmer OM, White CL (2022). The dual fates of exogenous tau seeds: lysosomal clearance versus cytoplasmic amplification. J Biol Chem.

[44] Brazill JM, Zhu Y, Li C, Zhai RG (2018). Quantitative cell biology of neurodegeneration in Drosophila through unbiased analysis of fluorescently tagged proteins using ImageJ. J Vis Exp.

[45] Kruger U, Wang Y, Kumar S, Mandelkow EM (2012). Autophagic degradation of tau in primary neurons and its enhancement by trehalose. Neurobiol Aging.

[46] Schaeffer V, Lavenir I, Ozcelik S, Tolnay M, Winkler DT, Goedert M (2012). Stimulation of autophagy reduces neurodegeneration in a mouse model of human tauopathy. Brain.

[47] Ichimura Y, Kirisako T, Takao T, Satomi Y, Shimonishi Y, Ishihara N (2000). A ubiquitin-like system mediates protein lipidation. Nature.

[48] Jipa A, Vedelek V, Merenyi Z, Urmosi A, Takats S, Kovacs AL (2021). Analysis of Drosophila Atg8 proteins reveals multiple lipidation-independent roles. Autophagy.

[49] Nezis IP, Simonsen A, Sagona AP, Finley K, Gaumer S, Contamine D (2008). Ref(2) P, the Drosophila melanogaster homologue of mammalian p62, is required for the formation of protein aggregates in adult brain. J Cell Biol.

[50] Pankiv S, Clausen TH, Lamark T, Brech A, Bruun JA, Outzen H (2007). p62/SQSTM1 binds directly to Atg8/LC3 to facilitate degradation of ubiquitinated protein aggregates by autophagy. J Biol Chem.

[51] Narasimhan S, Changolkar L, Riddle DM, Kats A, Stieber A, Weitzman SA (2020). Human tau pathology transmits glial tau aggregates in the absence of neuronal tau. J Exp Med.

[52] Martini-Stoica H, Cole AL, Swartzlander DB, Chen F, Wan YW, Bajaj L (2018). TFEB enhances astroglial uptake of extracellular tau species and reduces tau spreading. J Exp Med.

[53] Bai B, Vanderwall D, Li Y, Wang X, Poudel S, Wang H (2021). Proteomic landscape of Alzheimer’s disease: novel insights into pathogenesis and biomarker discovery. Mol Neurodegener.

[54] Sathe G, Albert M, Darrow J, Saito A, Troncoso J, Pandey A (2021). Quantitative proteomic analysis of the frontal cortex in Alzheimer’s disease. J Neurochem.

[55] Wang Z, Yu K, Tan H, Wu Z, Cho JH, Han X (2020). 27-Plex tandem mass tag mass spectrometry for profiling brain proteome in Alzheimer’s disease. Anal Chem.

[56] Higginbotham L, Ping L, Dammer EB, Duong DM, Zhou M, Gearing M (2020). Integrated proteomics reveals brain-based cerebrospinal fluid biomarkers in asymptomatic and symptomatic Alzheimer’s disease. Sci Adv.

[57] Bai B, Wang X, Li Y, Chen PC, Yu K, Dey KK (2020). Deep multilayer brain proteomics identifies molecular networks in Alzheimer’s disease progression. Neuron.

[58] Xu H, Chaturvedi R, Cheng Y, Bussiere FI, Asim M, Yao MD (2004). Spermine oxidation induced by Helicobacter pylori results in apoptosis and DNA damage: implications for gastric carcinogenesis. Cancer Res.

[59] Babbar N, Casero RA (2006). Tumor necrosis factor-alpha increases reactive oxygen species by inducing spermine oxidase in human lung epithelial cells: a potential mechanism for inflammation-induced carcinogenesis. Cancer Res.

[60] Capone C, Cervelli M, Angelucci E, Colasanti M, Macone A, Mariottini P (2013). A role for spermine oxidase as a mediator of reactive oxygen species production in HIV-Tat-induced neuronal toxicity. Free Radic Bio Med.

[61] Narayanan SP, Xu Z, Putluri N, Sreekumar A, Lemtalsi T, Caldwell RW (2014). Arginase 2 deficiency reduces hyperoxia-mediated retinal neurodegeneration through the regulation of polyamine metabolism. Cell Death Dis.

[62] Mathys H, Davila-Velderrain J, Peng Z, Gao F, Mohammadi S, Young JZ (2019). Single-cell transcriptomic analysis of Alzheimer’s disease. Nature.

[63] Wirth M, Benson G, Schwarz C, Kobe T, Grittner U, Schmitz D (2018). The effect of spermidine on memory performance in older adults at risk for dementia: a randomized controlled trial. Cortex.

[64] Schwarz C, Horn N, Benson G, Wrachtrup Calzado I, Wurdack K, Pechlaner R (2020). Spermidine intake is associated with cortical thickness and hippocampal volume in older adults. Neuroimage.

[65] Pekar T, Bruckner K, Pauschenwein-Frantsich S, Gschaider A, Oppliger M, Willesberger J (2021). The positive effect of spermidine in older adults suffering from dementia: first results of a 3-month trial. Wien Klin Wochenschr.

[66] Schroeder S, Hofer SJ, Zimmermann A, Pechlaner R, Dammbrueck C, Pendl T (2021). Dietary spermidine improves cognitive function. Cell Rep.

[67] Freitag K, Sterczyk N, Wendlinger S, Obermayer B, Schulz J, Farztdinov V (2022). Spermidine reduces neuroinflammation and soluble amyloid beta in an Alzheimer’s disease mouse model. J Neuroinflammation.

[68] Murray Stewart T, Dunston TT, Woster PM, Casero RA (2018). Polyamine catabolism and oxidative damage. J Biol Chem.

[69] Butterfield DA, Halliwell B (2019). Oxidative stress, dysfunctional glucose metabolism and Alzheimer disease. Nat Rev Neurosci.

[70] Bartolome F, Carro E, Alquezar C (2022). Oxidative stress in tauopathies: from cause to therapy. Antioxidants.

[71] Hofer SJ, Simon AK, Bergmann M, Eisenberg T, Kroemer G, Madeo F (2022). Mechanisms of spermidine-induced autophagy and geroprotection. Nat Aging.

[72] Sakamoto A, Terui Y, Uemura T, Igarashi K, Kashiwagi K (2020). Polyamines regulate gene expression by stimulating translation of histone acetyltransferase mRNAs. J Biol Chem.

[73] Mandal S, Mandal A, Johansson HE, Orjalo AV, Park MH (2013). Depletion of cellular polyamines, spermidine and spermine, causes a total arrest in translation and growth in mammalian cells. Proc Natl Acad Sci USA.

[74] Zwighaft Z, Aviram R, Shalev M, Rousso-Noori L, Kraut-Cohen J, Golik M (2015). Circadian clock control by polyamine levels through a mechanism that declines with age. Cell Metab.

[75] Puleston DJ, Buck MD, Klein Geltink RI, Kyle RL, Caputa G, O’Sullivan D (2019). Polyamines and eIF5A hypusination modulate mitochondrial respiration and macrophage activation. Cell Metab.

[76] Wang J, Li S, Wang J, Wu F, Chen Y, Zhang H (2020). Spermidine alleviates cardiac aging by improving mitochondrial biogenesis and function. Aging.

