# Supplementary material for: Reduction of spermine synthase enhances autophagy to suppress Tau accumulation
Source: Cell Death Dis. 2024 May 13;15(5):333. doi: 10.1038/s41419-024-06720-8 (PMC11091227; doi:10.1038/s41419-024-06720-8)
Supplement: Supplementary file 1 — Supplementary figures [file 41419_2024_6720_MOESM1_ESM.docx]

**Supplementary Information**

**Reduction of spermine synthase enhances autophagy to suppress Tau accumulation**

Xianzun Tao^1^, Jiaqi Liu^1^, Zoraida Diaz-Perez^1^, Jackson R. Foley^2^, Ashley Nwafor^2^, Tracy Murray Stewart^2^, Robert A. Casero Jr.^2^, R. Grace Zhai^1*^

^1^Department of Molecular and Cellular Pharmacology, University of Miami Miller School of Medicine, Miami, Florida, USA

^2^Sidney Kimmel Comprehensive Cancer Center, Johns Hopkins School of Medicine, Baltimore, Maryland, USA

*Corresponding author

Email: [gzhai@med.miami.edu](mailto:gzhai@med.miami.edu)

Phone: +1 (305) 2436316

Supplementary Figures: Page 2-6

**
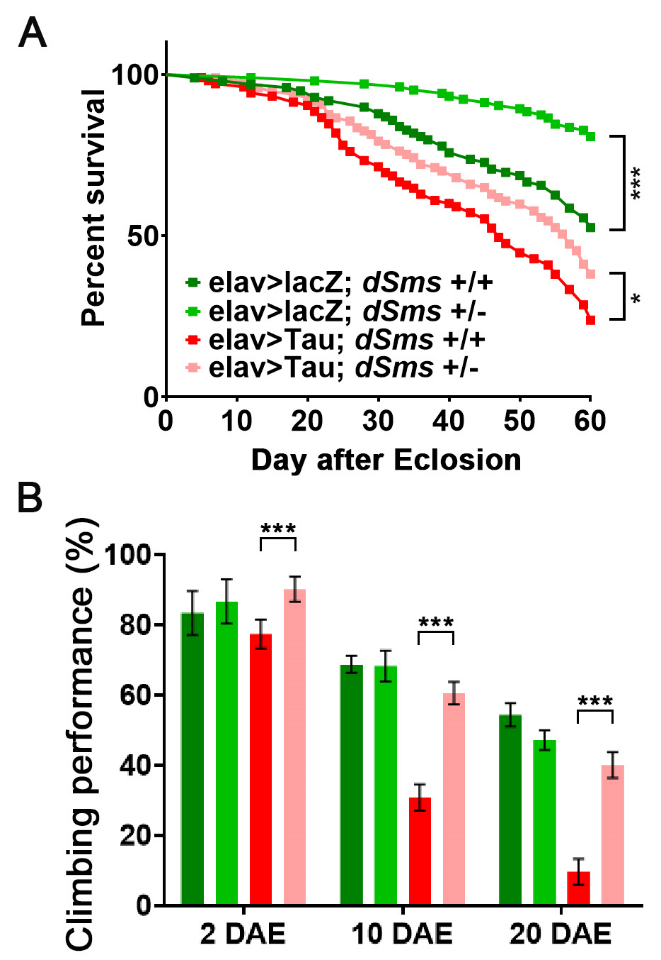
**

**Figure S1. SMS reduction extends lifespan and improves climbing performance in male Tauopathy flies.** (A) Lifespan of male flies with indicated genotype. n = 99, 104, 105, 97; Log-rank (Mantel-Cox) test. (B) Climbing performance of male flies with indicated genotype at indicated ages. n = 100, 100, 100, 100; two-way ANOVA Sidak’s multiple comparisons. Data represent mean ± SEM.

**
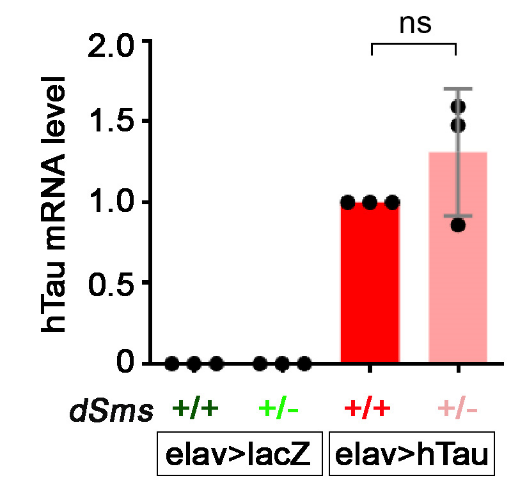
**

**Figure S2. SMS reduction does not significantly change the mRNA level of Tau in fly heads.** 30 heads of 10 DAE flies with indicated genotype were lysed with 500 uL of TRI Reagent (T9424, Sigma) using a bullet blender. RNAs were isolated following the manufacturer’s manual. cDNAs were reverse transcribed from the RNAs with a High-Capacity cDNA Reverse Transcription Kit (4367381, Applied Biosystems). The cDNA levels of Tau and RP49 (as a control) were measured with a qPCR kit (1725270, Bio-Rad). Tau mRNA levels (cDNA levels) were normalized with RP49 mRNA levels (cDNA levels). The relative Tau mRNA level from *dSms^+/+^* flies with Tau overexpression was set as 1. n = 3 (three batches of flies); Student’s t test. Data represent mean ± SEM. Primers used: CCAAGTGTGGCTCATTAGGCA (hTauF), CCAATCTTCGACTGGACTCTGT (hTauR), CTAAGCTGTCGCACAAATGGC (dRP49F), AACCGATGTTGGGCATCAGA (dRP49R).

**
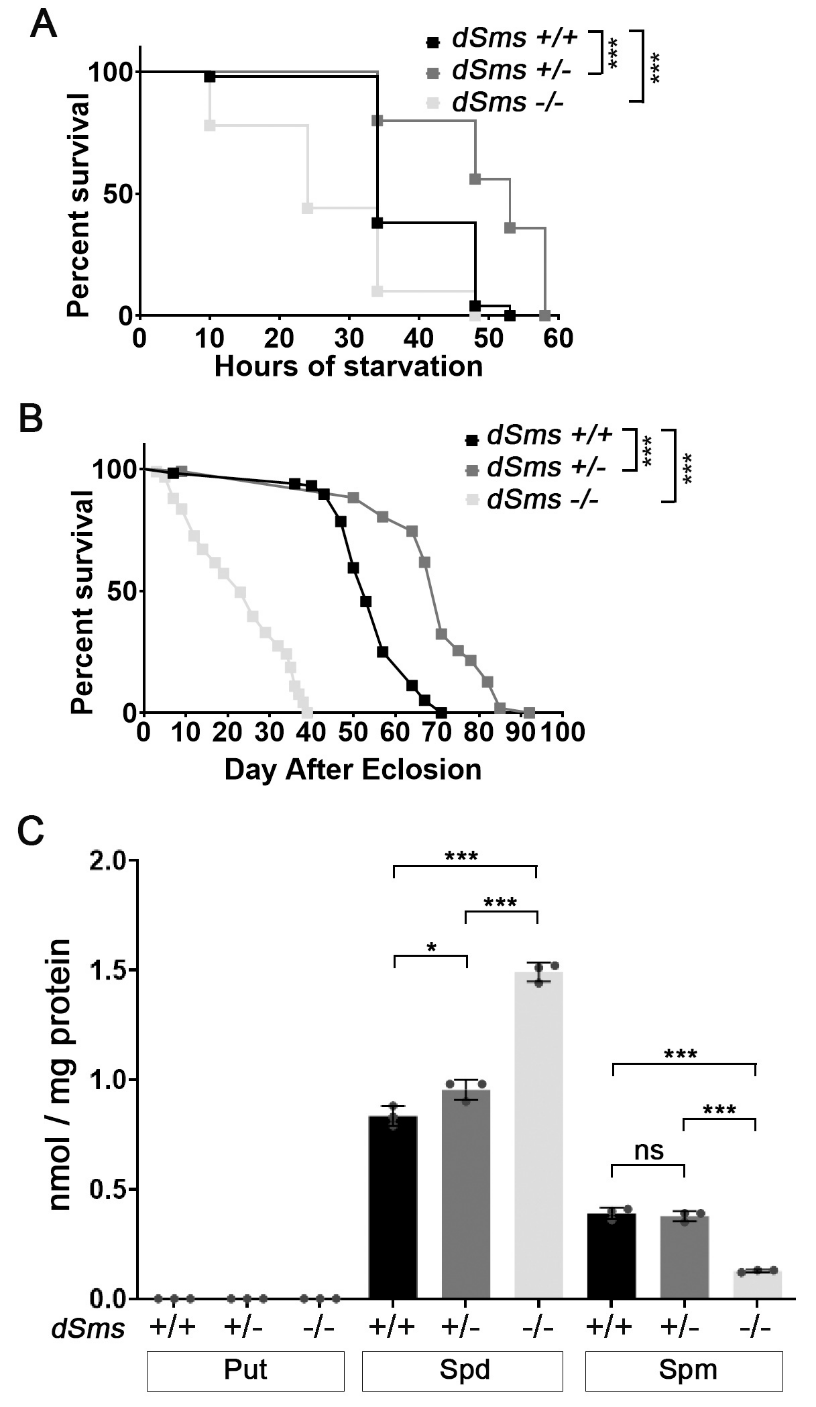
**

**Figure S3. SMS regulates autophagy function in male flies.** (A) Survival curve of 10 DAE male flies with indicated genotype under starvation. n = 50, 50, 50; Log-rank (Mantel-Cox) test. (B) Lifespan of male flies with indicated genotype. n = 116, 102, 91; Log-rank (Mantel-Cox) test. (C) Polyamine levels of 10 DAE male flies with indicated genotype. Each dot indicates a sample of homogenized mixture of 10 flies. n = 3; one-way ANOVA Tukey’s multiple comparisons. Data represent mean ± SEM. The measurement showed here was done together with that showed in our previous publication (Tao, X., et al., *JCI Insight*, 2022. 7(13)). The data of the control and *dSms^-/-^* flies are shared in these two studies.


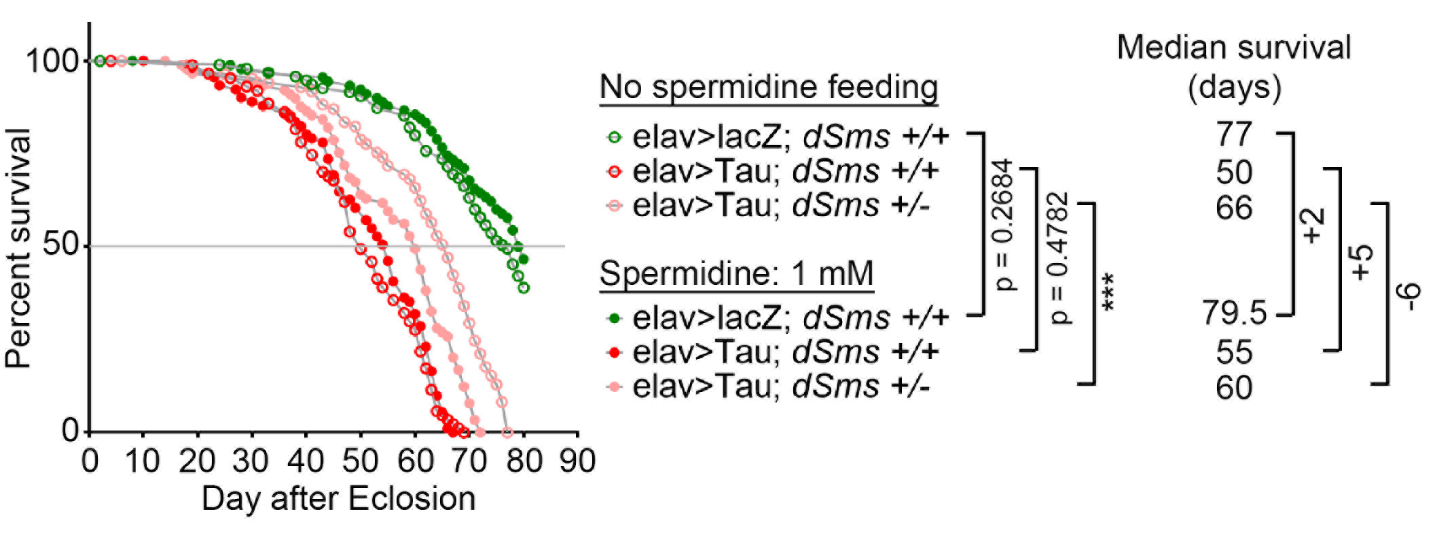


**Figure S4. Spermidine supplementation shows toxic effect on *dSms* ^-/-^** **flies with Tau overexpression.** Lifespan of flies with indicated genotype with or without 1 mM of spermidine in the food. n = 96, 88, 86, 91, 92, 90; Log-rank (Mantel-Cox) test. Of note, elav (C155) driver was used for lacZ or hTau expression in this experiment, which is different to the elav driver used in other experiments in this publication. The flies with hTau expression in this experiment live a little longer.

**
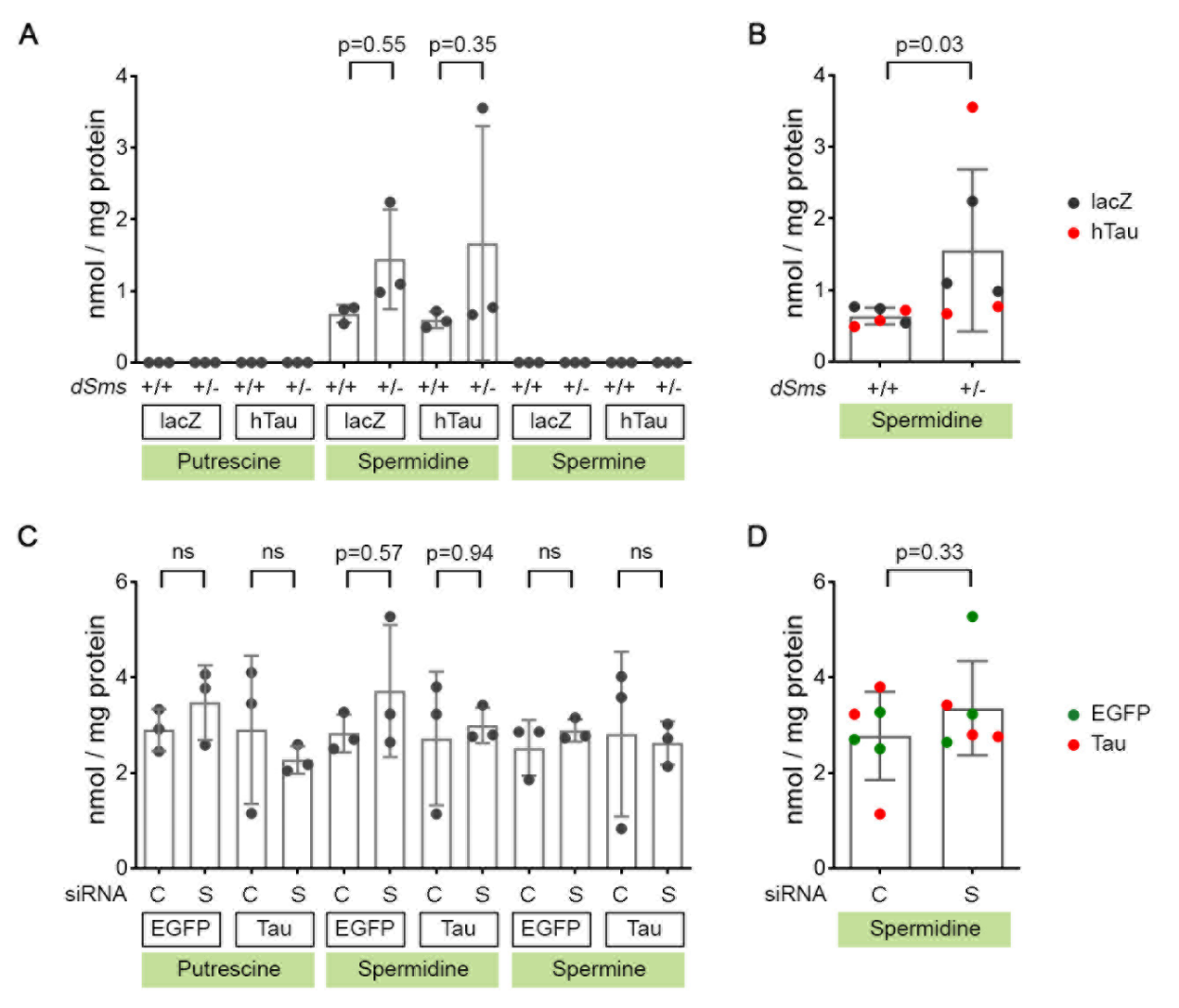
**

**Figure S5. Polyamine levels in fly heads or human cells with SMS reduction.** (A) Polyamine levels of 10 DAE female fly heads with indicated genotype. Each dot indicates a sample of homogenized mixture of 30 fly heads. Putrescine and spermine levels were under the detection limit. n = 3; two-way ANOVA Sidak’s multiple comparisons. (B) Analysis of spermidine levels in combined sample groups expressing either lacZ or hTau from (A). n = 6; Student’s t test. (C) Polyamine levels of SH-SY5Y cells with EGFP/Tau plasmids and Control (C) /SMS (S) siRNA transfection. n = 3 (three experiments); two-way ANOVA Sidak’s multiple comparisons. ns, not significant. (D) Analysis of spermidine levels in combined sample groups expressing either EGFP or Tau from (C). n = 6; Student’s t test. Data represent mean ± SEM.
