# Supplementary figures and images for: Reduction of spermine synthase enhances autophagy to suppress Tau accumulation
Source: Cell Death Dis. 2024 May 13;15(5):333. doi: 10.1038/s41419-024-06720-8 (PMC11091227; doi:10.1038/s41419-024-06720-8)

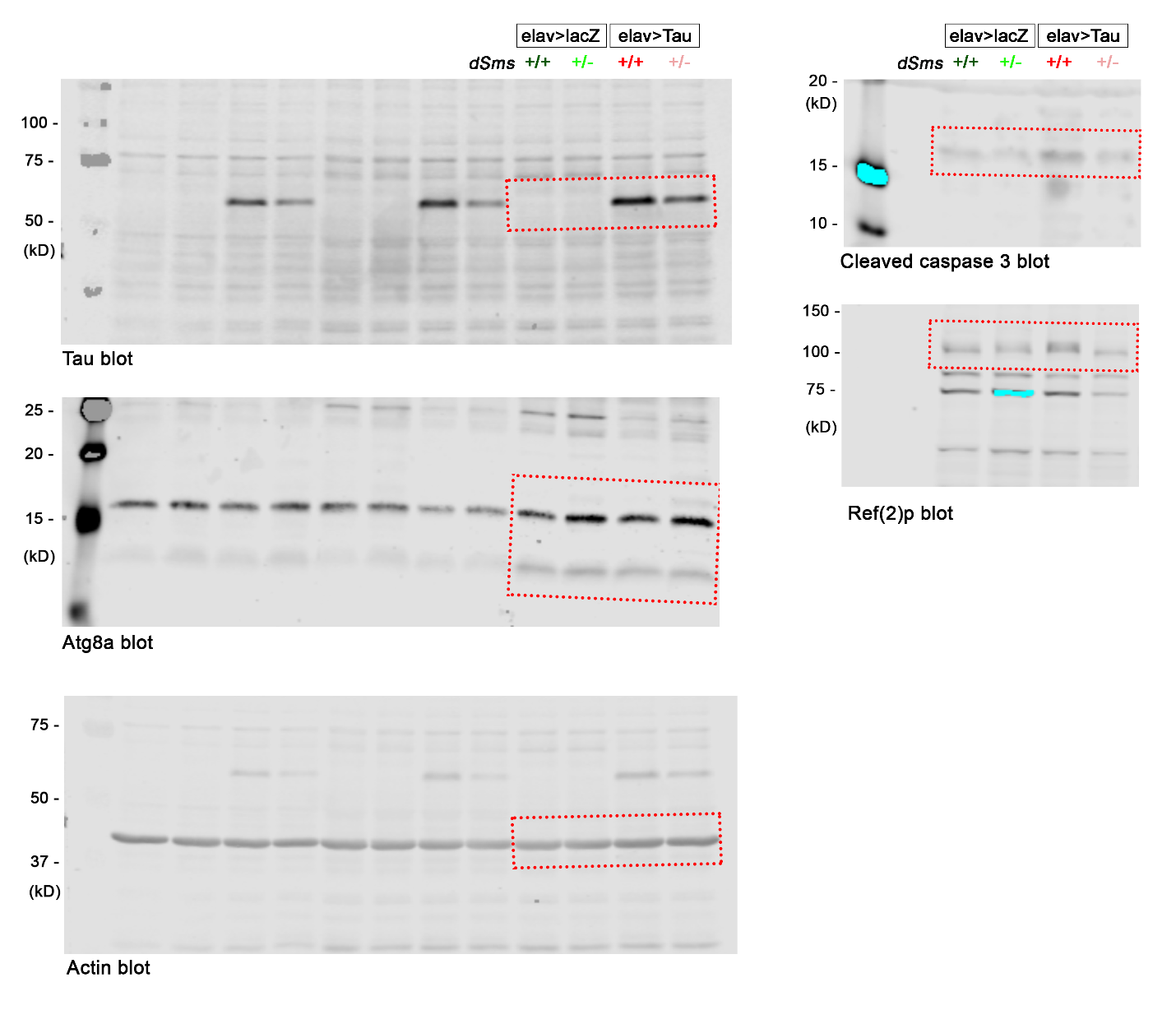


Original blots for Figure 1F.


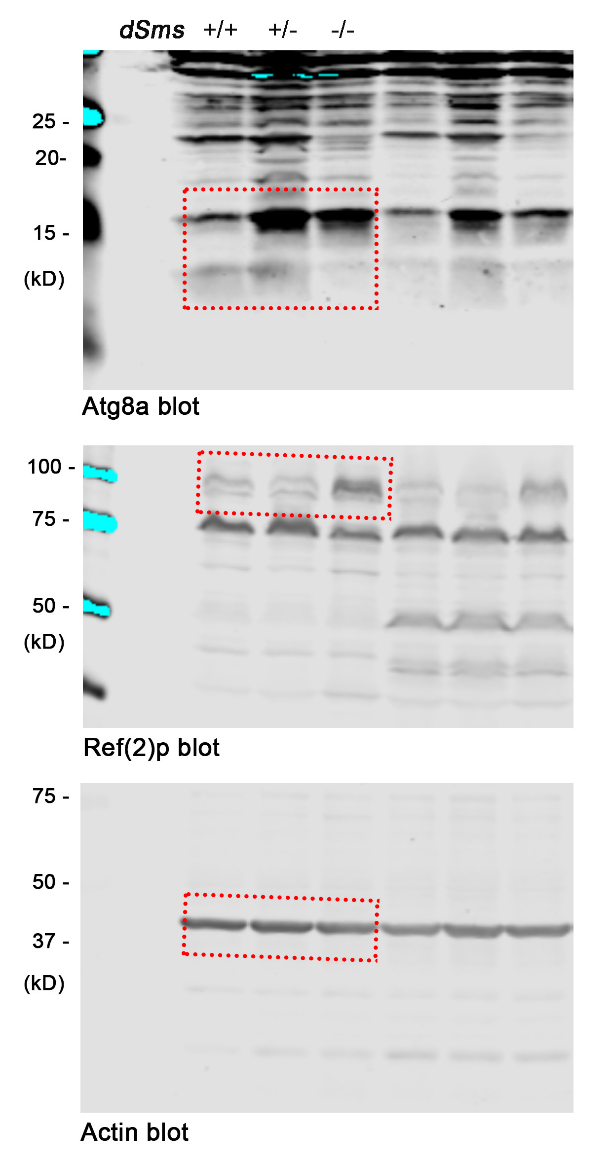


Original blots for Figure 2A.


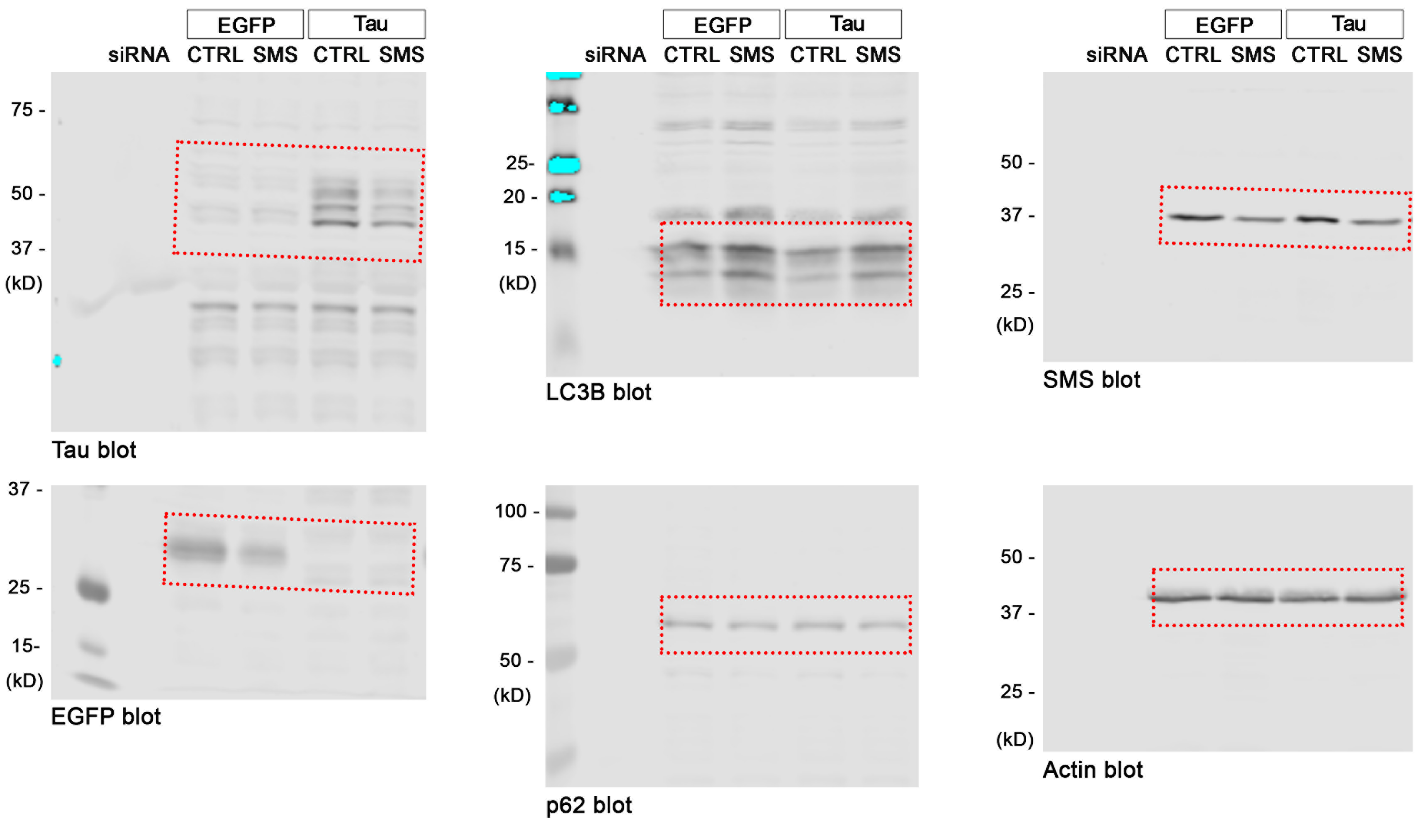


Original blots for Figure 4A.

Supplement: Supplementary file 2 — Original western blots [file 41419_2024_6720_MOESM2_ESM.docx]
